# An 18-day, 3 °C cold treatment effectively kills *Ceratitis capitata* (Diptera: Tephritidae) in kiwifruit (*Actinidia* spp.)

**DOI:** 10.1093/jee/toad242

**Published:** 2024-01-23

**Authors:** Samuel D J Brown, André M Bellvé, Karina Santos, Cristian E Baldassarre, Emma Mansfield, Vineeta Bilgi, Elodie M G Urlacher, Jessica C Devitt, Lisa E Jamieson

**Affiliations:** The New Zealand Institute for Plant and Food Research Limited, Mount Albert Research Centre, Auckland, New Zealand; The New Zealand Institute for Plant and Food Research Limited, Mount Albert Research Centre, Auckland, New Zealand; The New Zealand Institute for Plant and Food Research Limited, Mount Albert Research Centre, Auckland, New Zealand; The New Zealand Institute for Plant and Food Research Limited, Mount Albert Research Centre, Auckland, New Zealand; Department of Primary Industries and Regional Development, South Perth, WA, Australia; Department of Primary Industries and Regional Development, South Perth, WA, Australia; Ministry for Primary Industries Manatū Ahu Matua, Wellington, New Zealand; Ministry for Primary Industries Manatū Ahu Matua, Wellington, New Zealand; The New Zealand Institute for Plant and Food Research Limited, Mount Albert Research Centre, Auckland, New Zealand

**Keywords:** post-harvest, disinfestation, phytosanitary, quarantine treatment

## Abstract

A series of experiments were carried out to develop a phytosanitary disinfestation protocol to kill *Ceratitis capitata* (Weidemann) (Mediterranean fruit fly, Diptera: Tephritidae) in ‘Hayward’ kiwifruit (*Actinidia deliciosa* (A. Chev.) C.F. Liang and A.R. Ferguson) and ‘Zesy002’ kiwifruit (*Actinidia chinensis* Planch.). Experiments on 4 immature life stages (eggs and 3 larval instars) with treatment durations of between 5 and 18 days showed that third instars were the most tolerant to temperatures around 3 °C, with the lethal time to 99.9968% (probit 9) mortality (LT_99.9968_) estimated to be 17.3 days (95% confidence interval (CI) 16.4–18.2). Larvae reared and treated in ‘Zesy002’ were significantly more susceptible to cold treatment than those reared in ‘Hayward’. A large-scale trial testing a disinfestation protocol of 3 ± 0.5 °C for 18 days treated over 500,000 third-instar *C. capitata* with no survivors. These results demonstrate that a cold treatment of 3.5 °C or below for 18 days induces *C. capitata* mortality in kiwifruit at a rate that exceeds 99.9968% with a degree of confidence greater than 99%.

## Whakarāpopoto

He momo ngaro nō Awherika te *Ceratitis capitata* (Wiedemann) (Diptera: Tephritidae: Dacinae). Engari, kua hōrapa whānui tēnei momo ki Ūropi, Amerika ki te Tonga, Hawai’i me tae atu hoki ki Ahitereiria ki te Hauāuru. Kai ai āna pūhouhou i te maha o ngā hua, tae ana ki te huakiwi (*Actinidia deliciosa* (A. Chev.) C.F. Liang me A.R. Ferguson me *Actinidia chinensis* Planch.). Kāore te *C. capitata* e noho ki Aotearoa, engari kua rokohanga tēnei momo ki konei i mua. Ki te urutomo te momo nei ki Aotearoa, ka pākino rawatia te ahumāra o Aotearoa. Otiia, mate ai āna pūhouhou i te makariri. Kua whakamahi mātou i ētahi whakamātautau ki te rēkoata raraunga-ā-mate mō ngā pūhouhou ō *C. capitata* i roto i te huakiwi. Ki te whakamahi tatou i te pārongo nei, ka whakahekengia te whakaaweawe o *C. capitata* ki te mea ka tae mai te momo nei ki Aotearoa.

## Introduction

*Ceratitis capitata* (Weidemann) (Diptera: Tephritidae: Dacinae, Mediterranean fruit fly) is a major pest of horticultural crops, with a broad range of hosts. Female flies lay their eggs in ripening fruit, in which the larvae develop through 3 instars over about 2 weeks, depending on temperature (Shoukry and Hafez 1979, Ricalde et al. 2012). Larvae feed on fruit pulp by using associated bacteria to break down the fruit and release nutrients, which are then ingested by the larvae (Bateman 1972, Fletcher 1987). Upon maturity, pre-pupal larvae leave the fruit to pupate in the soil. Adults emerge a few days later and reach reproductive maturity in about 5 days (Arita 1982). Larval damage to fruit renders it inedible, causing significant damage to the horticultural industries where it is present. The host range of *C. capitata* is extensive, with 66 suitable host fruit species in Western Australia (Woods et al. 2005) and 60 host fruit species on the island of Hawai’i (Liquido et al. 1990).

Originating in sub-Saharan Africa (Ruiz-Arce et al. 2020), *C. capitata* underwent rapid dispersal in the 19th century, assisted by unregulated global trade in fruit (De Meyer et al. 2002, Karsten et al. 2015). In Australia, it was first found in Perth, Western Australia, in 1895, then established in Tasmania, New South Wales, and Victoria (Bonizzoni et al. 2004). These Eastern populations declined and died out in the 1940s, making Western Australia the only Australian state to have *C. capitata* at present (Bonizzoni et al. 2004). Incursions of *C. capitata* into New Zealand occurred in 1907 (Napier and Blenheim) and 1996 (Auckland) (Cockayne 1907, Holder et al. 1997). There have been additional incursions of other fruit fly species, most recently *Bactrocera tryoni* (Froggatt) (Queensland fruit fly) and *B. facialis* (Coquillett) (Tongan fruit fly) in Auckland in 2019 (Pather et al. 2019). In all cases, the detection of fruit flies has initiated a commendably rapid and sufficient response by governmental agencies, which resulted in the successful eradication of these incursions. No economically important tephritid fruit fly species have established populations in New Zealand. Consequently, New Zealand’s horticultural industries have substantially greater fruit production and access to overseas markets, and they do not incur the costs of fruit fly management.

Kiwifruit are a significant crop in New Zealand, with a growing area of nearly 13,000 ha and an export value of $2.7 billion NZD ($1.6 billion USD) in 2021 (Aitken and Warrington 2021). The major cultivars grown are ‘Hayward’ (*Actinidia deliciosa* (A. Chev.) C.F. Liang and A.R. Ferguson) and ‘Zesy002’ (*Actinidia chinensis* Planch.) (Ferguson 2013, Aitken and Warrington 2020). In Greece and Argentina, *C. capitata* has been recorded in low numbers from kiwifruit (Papadopoulos et al. 2001, Segura et al. 2002). However, Mediterranean fruit flies preferentially attack fruits such as plums, feijoas, peaches, apples, and pears in these countries (Papadopoulos et al. 2001, Segura et al. 2002).

Any future fruit fly incursion or establishment in New Zealand would significantly impact horticultural industries, disrupting exports and requiring implementation of in-orchard control practices. Pre-export phytosanitary treatments are an essential tool for mitigating these risks. Many economically significant Tephritidae are susceptible to low temperatures (below 5 °C) (Myers et al. 2016), making cold treatments a feasible option for treating fresh produce possibly infested with fruit flies. These treatments are readily incorporated into the supply chain, as low temperatures are routinely used to store and transport kiwifruit (Burdon and Lallu 2011). An effective treatment protocol would allow exports to continue unhindered through any future incursion or establishment of *C. capitata* in New Zealand, mitigating the economic impact such an event may have.

We demonstrate that an 18-day, 3 °C cold treatment is an effective disinfestation protocol for *C. capitata* in kiwifruit. Evidence for the efficacy of this treatment is provided by a series of experiments that developed and tested such a protocol.

## Methods

### Fruit Fly Colony and Mass-rearing Methods

*Ceratitis capitata* eggs were collected from a captive colony maintained by the Department of Primary Industries and Regional Development (DPIRD), South Perth, Western Australia. The colony was initially established in April 1983 from infested citrus fruits from Carnarvon. To maintain genetic fitness, the colony has been regularly supplemented by the inclusion of wild flies collected from locations throughout Western Australia on various host fruits, including adding flies from Gingin, Perth, and Manjimup in 2017, 2019, and 2020. Adult flies (50: 50 sex ratio) were held in 4 cages of 200 cm (length) × 150 cm (height) × 40 cm (width), with approximately 250,000 flies per cage. Colonies were held in rooms controlled at 26 ± 1 °C and 65 ± 5% relative humidity (RH) and were kept in the dark except when illuminated with halogen lamps to induce oviposition. Adult flies were provided with water, crystalline white sugar, and yeast hydrolysate from emergence. Eggs were collected by placing a 200-cm length of PVC guttering filled with 4 cm of water underneath the muslin sides of the fly colony cages. Flies oviposited through the muslin and eggs fell into the water. After 1–2 h, depending on the number of eggs required, eggs were washed out of the guttering using tap water. Colonies were cultured weekly using a paper-based diet with nutrients, including protein and sugar (De Lima et al. 2017). New cages were established every week.

### Fruit Used for Experiments

Insecticide residue-free kiwifruit were used for cold treatment experiments. ‘Hayward’ and ‘Zesy002’ kiwifruit (fruit weights ranging from 108 to 180 g) were sourced from orchards in the Bay of Plenty and Auckland regions in New Zealand. Fruits were shipped to DPIRD and stored at 1 °C until required. The fruit was about 7–9 months post-harvest when experiments commenced but still in good condition. All experiments were conducted in DPIRD’s South Perth disinfestation facilities in Perth, Western Australia, in 2018, 2019, and 2022. Both kiwifruit cultivars were used in cold tolerance experiments conducted in 2018 and 2019, but only ‘Hayward’ was used for the confirmatory large-scale trials in 2022.

### Infestation Protocol

All kiwifruit were artificially infested by boring a hole 25 mm deep into the calyx end of the fruit using a cork borer 5 mm in diameter. A second hole was bored into the cheek of the fruit so the 2 holes met at their lowest point. Two holes were made to promote drainage of fluid produced by larval activity and minimize drowning. An average of 90 fruit fly eggs (95% CI of 81.9–98.1 eggs) suspended in an agar solution (1.7 g/L with 0.105 g/L benzalkonium chloride delivered as 700 µL of Hy-Clor algaecide, HY-CLOR Australia Pty Ltd, Glendenning, Australia) were pipetted into the hole. After infestation, the holes were closed with balls of polyester wadding and fruits were housed in 10-L containers directly on a thin layer of sand. Boxes were held in a controlled temperature room set at 26 °C and 70% RH prior to cold treatment to allow larvae to develop to the desired stage for testing.

### Life History Trials

**Fig. 1.**
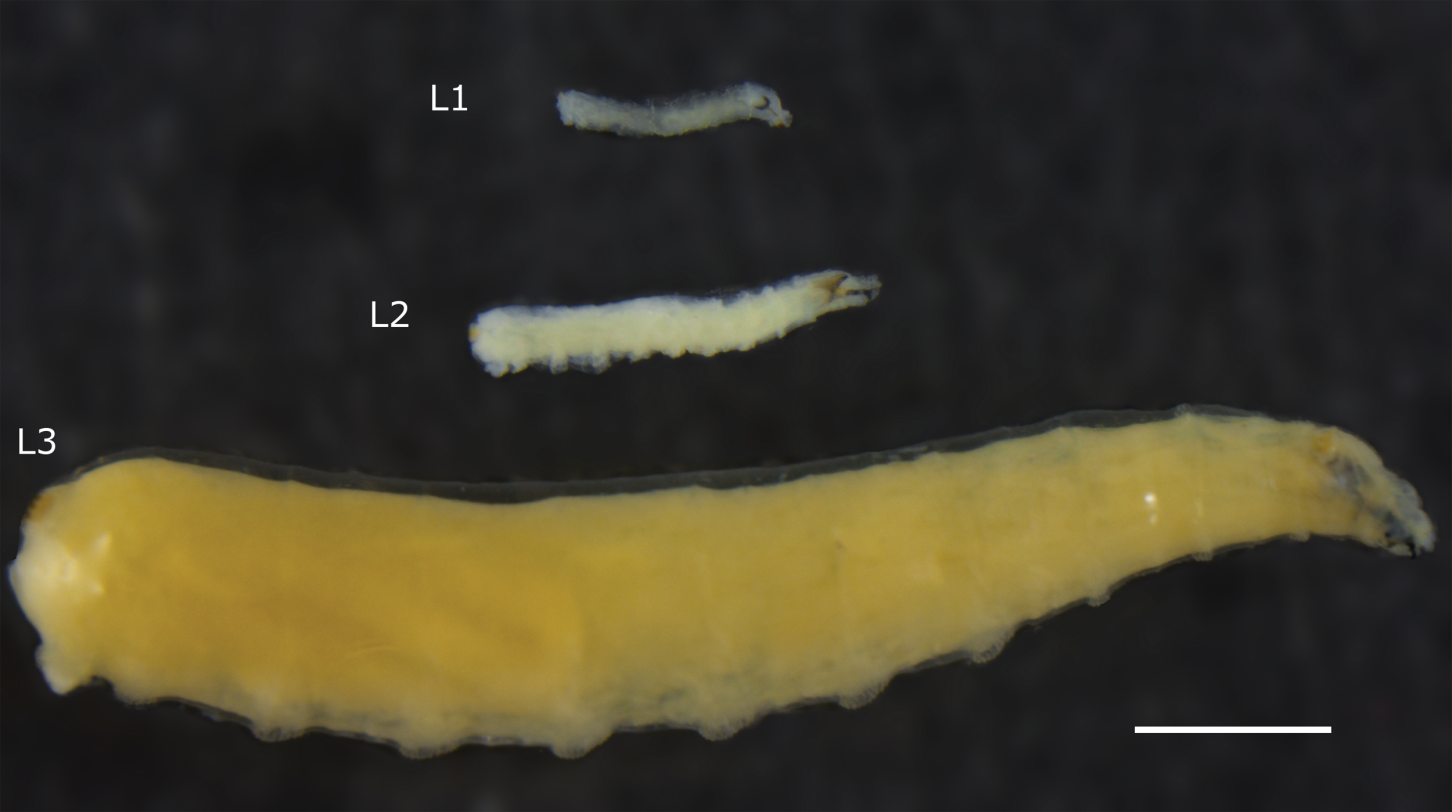
Mediterranean fruit fly (*Ceratitis capitata*) larval instars showing size differences. Top to bottom: first instar (L1; 3 days old), second instar (L2; 5 days old), and third instar (L3; 7 days old). Scale bar = 1 mm.

**Fig. 2.**
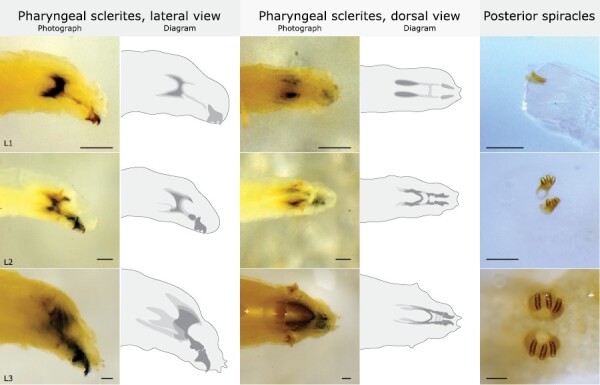
Morphological characters distinguishing Mediterranean fruit fly (*Ceratitis capitata*) larval instars. Scale bars = 0.1 mm. L1 = first instar. L2 = second instar. L3 = third instar.

To examine the developmental rates of *C. capitata* in kiwifruit, 48 fruits/cultivars were infested per replicate, with 3 replicates infested on consecutive days. After infestation, the kiwifruit were housed individually in 1-L plastic containers (172 × 120 × 71 mm, Chanrol, Chanrol Pty Ltd, Sydney, Australia) lined with a thin layer of sand. Containers were placed in a controlled temperature room set to 26 °C, 14:10 L:D photoperiod, and 70% RH. Each day, for 10 days post-infestation, 3 fruits/cultivars/replicates were randomly selected and dissected to count the number of each immature life stage present. The life stages of the recovered larvae were ascertained using morphological and behavioral features (Table 1, Figs. 1 and 2).

**Table 1.** Characteristics for distinguishing Mediterranean fruit fly (*Ceratitis capitata*) larval instars

Larval life stage	Key characters
First instar	**Size:** 0.8–1.3 mm, about the same size as an egg. **Coloration:** Nearly transparent, cloudy. **Pharyngeal sclerite:** Subrectangular in lateral view, short and disconnected in dorsal view. **Behavior:** Sluggish, wriggles slowly, sinks in water.
Second instar	**Size:** 1.7–3.7 mm **Coloration:** Anterior half translucent, posterior half opaque, often with a yellowish tinge (likely diet-dependent). **Pharyngeal sclerite:** Anvil-shaped in lateral view, forceps-like in dorsal view. **Behavior:** Active, remains submerged.
Late second instar	Approximately the same size as early third instars, but skin appears much tighter (like a sausage that is about to burst). Second pair of pharyngeal sclerites visible. Remains submerged.
Early third instar	Approximately the same size as late second instars, but skin very loose and saggy. Single set of pharyngeal sclerites present. Floats with spiracles at surface of water.
Third instar	**Size:** 3.1–7.4 mm **Coloration:** Totally opaque white, evenly distributed fat and muscle tissues. **Pharyngeal sclerite:** Boat-shaped in lateral view (lacking ventral posterior lobe), forceps-like in dorsal view. **Behavior:** Very active, floats with posterior spiracles on the surface of the water.

### Cold Tolerance Experiments

Experiments to evaluate the relative cold tolerance of the 4 immature life stages were conducted in 2018. In these experiments, fruits were infested at different times in advance of treatment to allow larvae to develop to the required life stage: 7 days for third instars, 5 days for second instars, 3 days for first instars, and 1 day for eggs. These times were chosen from life history studies of *C. capitata* conducted on ‘Hayward’ and ‘Zesy002’ kiwifruit prior to the commencement of these experiments.

For the cold tolerance experiments, 15 fruits/time durations/life stages/cultivars were infested in each replicate and housed in 3 boxes. Four replicates were carried out, being infested on 2 occasions, and these were treated at 3 °C in 2 separate cold rooms. Three boxes/life stage/cultivar/replicate were removed at each of 5, 6, 7, 8, and 10 days after the start of treatment. At each treatment point, boxes were removed into 26 °C, 14:10 L:D photoperiod, and 70% RH until they were assessed for pupal emergence.

In 2019, additional experiments were carried out to test longer treatment times on third-instar larvae only, these being the most tolerant life stage inferred from the 2018 cold tolerance experiments. In each replicate, 50 fruits/time durations/cultivars were infested and housed in 5 boxes; an additional 10 infested fruits were set aside for dissection when treatments started. Four replicates were prepared on consecutive days in October 2019, with 810 fruits infested in each replicate. Five boxes/cultivar/replicate were removed at each of 8, 10, 11, 13, 14, 16, and 18 days after being treated at 3 °C and held for a further 9 days for completion of larval development.

### Confirmatory Dissections

To ensure that the correct life stage was treated, additional fruits infested at the same time as treated fruits were removed for dissection at the commencement of cold treatments. The life stages of the recovered larvae were ascertained using morphological and behavioral features (Table 1, Figs. 1 and 2). Dissections were preferentially completed while the larvae were still alive on the day treatments started. When this was not possible, fruits were frozen on the day that treatment started and dissected at a later date.

### Pupal Assessments and Mortality Estimation

Both cold tolerance experiments assessed survival by counting the pupae in each box after 16 days + treatment duration. Assessments involved cutting through the fruit remains in each box to locate surviving larvae. Any live larvae discovered were placed into a box with dry sand, along with the fruit portion they were found in; these were retained for a further 2 days for pupation. After the fruit had been processed, the sand was washed through a 1.0-mm sieve stacked on top of a 125-µm sieve to retrieve and count the pupae.

Pupal counts were converted to proportional mortality using an estimated total based on the mean number of pupae retrieved from control boxes for each combination of cultivar, replicate, and life stage.

### Cold Treatment Facilities

In both cold tolerance experiments, all infested fruits for each replicate were placed into cold storage on the same day, except for the control (0-day treatment) boxes, which were retained at 26 °C. The 2 cold stores (CSs) used were 4.33 m × 3.78 m × 2.13 m (34.90 m^3^ total volume), adjacent to each other in a block of 3. Both CSs were fitted with roller doors and were cooled using a Copeland CCH 250 air-cooled condensing unit with R22 refrigerant and a Patton BL 28 low-profile unit cooler (Patton, Osborne Park, WA, Australia). Air was circulated through the room by two 300-mm 5-bladed propeller fans, making an average airflow of 960 L s^−1^ (De Lima et al. 2017).

### Temperature Measurement

Temperatures within each CS were logged every 30 min with a Grant Squirrel meter/logger (Model SQ2020-2F8, Cambridge, England), fitted with 16 type-U thermistor temperature probes with accuracy ± 0.01 °C in the range of −25 °C to + 125 °C: 4 air probes were located in the corners of the CS, 1 air probe was located behind the blower, and 1 air probe at the door, while 10 probes were inserted into the core of uninfested fruits, which were divided between the 2 sides of the CS (5 fruits/side). Probes were calibrated using an ice bath (ASTM E562-11 2016) before treatments commenced and after they had concluded. All probes were placed into an ice bath and temperature measurements were made every 10 s for between 10 and 13 min. The mean calibration factors were −0.09 ± 0.01 °C and −0.09 ± 0.02 °C (mean ± standard error) for fruit and air probes, respectively.

### Large-Scale Trials

A large-scale trial was conducted to test the prospective cold treatment protocol of 3 °C for 18 days. The greater cold tolerance of *C. capitata* third instars on ‘Hayward’ kiwifruit, and the greater number of pupae recovered from this cultivar, led us to use only ‘Hayward’ kiwifruit in these trials. Forty pallets of ‘Hayward’ kiwifruit (weight range 108–128 g), containing 100 cartons per pallet (86–92 pieces of fruit per carton), were sourced from 1 orchard in Te Puke, New Zealand. The fruit was harvested in May 2021 and shipped to DPIRD in September 2021. After arrival, the fruit was stored at 1 °C until trials commenced in February 2022.

Using the methods described above, kiwifruit were artificially infested with an average of 105 fruit fly eggs (95% CI = 101.7–107.9 eggs). After infestation, infested fruits were held at 26 °C, 14:10 L:D photoperiod, and 70% RH for 7 days to allow larvae to develop to the third instar. Three replicates were prepared on consecutive days; each replicate consisted of 3,400 fruits infested for the 18-day treatment time, 180 for 2 shorter treatment durations of 8 and 10 days, 200 for untreated controls, and 10 for confirmatory dissections, making a total of 3,770 fruits infested for each replicate. Over the course of the large-scale trial, a total of 11,310 fruits were infested.

An additional 30 kiwifruits were infested and kept at 26 °C, 14:10 L:D photoperiod, and 70% RH until pupation as an ancillary trial to estimate the number of pupae expected to emerge from a single kiwifruit and to provide an additional avenue to estimate the number of insects treated during the trial.

Infested fruits for each replicate were placed into cold storage on the same day, except for the control (0-day treatment) fruits, which were retained at 26 °C for the duration of the experiment. Infested fruits were distributed among 8 pallets of uninfested filler ‘Hayward’ kiwifruit, ensuring that realistic cool-down periods were experienced in these semi-commercial trials. A total of 4–6 infested kiwifruit were placed on 279 × 228 × 30 mm Plix open cell absorbent polystyrene trays (TPM Packaging) or 233 × 155 × 55 mm cardboard food trays (CastAway, MPM Marketing Services, Brisbane) inside cardboard boxes filled with uninfested kiwifruit. These boxes were the modular bulk (MB) (300 mm wide × 400 mm long × 187 mm high) standard packaging type used by Zespri for shipping kiwifruit. Pallets were made up of 7 or 8 layers, each made up of 10 MB boxes. No gap was present in the middle of the pallet. All pallets were strapped twice with 4 corner boards in place. Infested fruits intended for removal at 8 and 10 days after treatment were placed in plastic tote trays lined with a layer of sand and placed on top of the pallets nearest the door once all pallets were loaded. Treatments were deemed to have started when over half of the fruit probes measured below 3.5 °C.

At 8 and 10 days after treatments began, 5 sand-lined tote trays/replicate were removed into 26 °C, 14:10 L:D photoperiod, and 70% RH and held for 9–14 days after treatment to allow any larval development to proceed to pupation. The sand was washed through a large 1.0-mm rectangular sieve, and the pupae were counted.

After the 18-day treatment, the pallets were broken down and the infested fruits were removed from the MB boxes. Because there were a large number of control and treatment fruits, these were held for pupation in wire basket towers in a laboratory at 26 °C, 14:10 L:D photoperiod, and 70% RH. Control fruits were held for pupation in a separate laboratory from treated fruits. Towers were comprised of 5 wire baskets stacked on each other, separated by strong metal rods in a cross design to keep the baskets separated, allow air circulation, and prevent the fruit from being squashed. A large metal funnel was placed below the baskets, emptying into a deep-square tray filled with sand that fitted snugly underneath the tower. Upon emergence, larvae fell into the sand and pupated. The tower was covered with a large muslin bag to ensure pupae remained inside and to prevent the decaying fruit from being colonized by drosophilid flies. The sand from each tower was replaced every few days. On replacement, the old sand was washed through a 1.0-mm sieve and the pupae were retrieved and frozen for subsequent counting. Towers were in operation for 4 weeks after treatment to allow any living larvae time to pupate. All assessments were completed by 2 March 2022.

### Statistical Analysis

Generalized linear mixed models were used to analyze the mortality of immature *C. capitata* after different cold treatment durations.

One model was fitted with data only from the 2018 experiments to evaluate the relative cold tolerance of the 4 immature life stages tested. The fixed effects for this model were a life stage and a life stage: treatment time interaction, with replicate and a replicate: cultivar interaction as random effects.

Another model with a similar structure was fitted to a combined dataset of the 2018 and 2019 experiments. By combining these datasets we evaluated whether the increased treatment range (i.e., testing up to 18 days in cold storage) altered predictions for our CIs around these estimates.

Separate models were fitted for the data from the 2 kiwifruit cultivars to derive cultivar-specific lethality estimates. These models had fixed effects for a life stage and a life stage: treatment time interaction, with a random effect accounting for the variation inherent between replicates and years. The best model for ‘Hayward’ used a logit link function, whereas the best model for ‘Zesy002’ used a probit link function.

Model assumptions of binomial dispersion were checked using simulation-based tests (Hartig 2020). These showed that the extra-binomial dispersion in the data was adequately mitigated by incorporating a beta-binomial dispersion parameter into the models. These dispersion parameters had different intercepts for each life stage and slopes as a function of treatment time. Including the dispersion parameter correctly accounted for the error in the model and provided realistic CIs relative to binomial models that could not account for this overdispersion and produced much wider CIs around estimates from the model.

We calculated lethality estimates for various mortality levels, being 50%, 95%, 99%, and 99.9968% mortality. The 95% CIs around these estimates were calculated using Fieller’s formula (Fieller 1954). Assurance that the large-scale trials detected these mortality levels with a high degree of confidence was provided by using calculations formulated by Couey and Chew (1986).

Estimates of the total number of specimens treated in the large-scale trial were extrapolated from evidence of the numbers of specimens recovered from the controls by calculating the 2-sided 95% CI of the mean and multiplying by the factor by which the large-scale trial was larger than the controls. Explicitly, this involved the calculation:


$$\begin{array}{l} n_{treated}=\left(\mu \pm \frac{s}{\sqrt{r}}\;\times \;t_{0.975,\;r-1}\right)\times \;\frac{n_{l}}{n_{c}} \end{array}$$

Where *μ* = the overall mean from the controls, *s* = the standard deviation of the controls, *r* = the number of replicates, $t_{0.975,\;r-1}$is the 95.7^th^ percentile of the *t* distribution with *r* − 1 degrees of freedom, $n_{l}$= the number of fruits infested in the large-scale trials, and $n_{c}$= the number of fruits infested in the control replicates. This differs subtly from the calculation recommended by Wright et al. (2023), who used the lower limit of a single-sided 95% CI. It also differs from International Plant Protection Convention (IPPC) recommendations (IPPC 2023, p 113) by using the standard error of the mean to calculate the CI, instead of the standard deviation.

All analyses were conducted in R (version 4.2.2.; R Core Team 2023) using a RStudio interface (version 2022.12.0 + 353 ‘Elsbeth Geranium’). Data manipulations were performed with dplyr (version 1.0.10), janitor (version 2.10), and stringr (version 1.5.0; Wickham 2022). Statistical models were fitted using glmmTMB (version 1.1.5; Brooks et al. 2017), with dispersion checks conducted using DHARMa (version 0.4.6; Hartig 2020). Estimated mortality times and associated CIs were calculated using qra (version 0.2.7). Data visualizations were produced using ggplot2 (version 3.4.0) and viridis (version 0.6.2).

## Results

### Life History of *C. capitata* in Kiwifruit

**Fig. 3.**
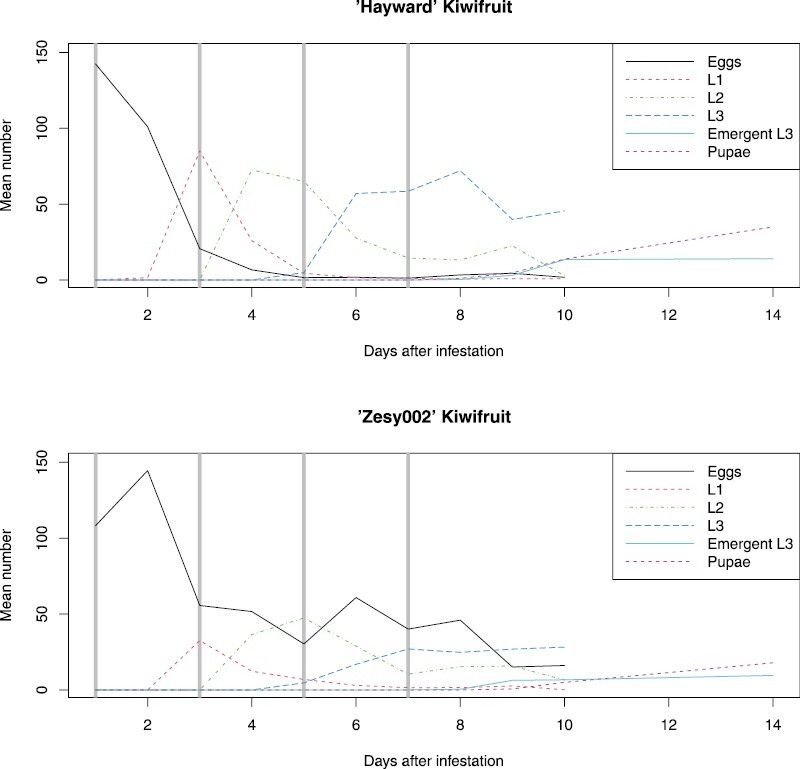
Mean numbers of immature life stages of Mediterranean fruit fly (*Ceratitis capitata*) present in fruits up to 10 days after infestation at 26 °C and 70% relative humidity. Vertical lines indicate chosen infestation periods. L1 = first instars. L2 = second instars. L3 = third instars. Emergent L3 = third instars which have left the fruit to search for a place to pupate.

Larval development in both kiwifruit cultivars proceeded at the same rate, though there were substantially reduced egg hatch rates in ‘Zesy002’ kiwifruit compared to ‘Hayward’. In both cultivars, eggs began to hatch 2 days after infestation. First instars were the predominant life stage after 3 days, second instars after 5 days, and third instars after 7 days (Fig. 3). Emergent third instars and pupae started to appear from 9 days after infestation.

### Relative Cold Tolerance of Immatures

**Fig. 4.**
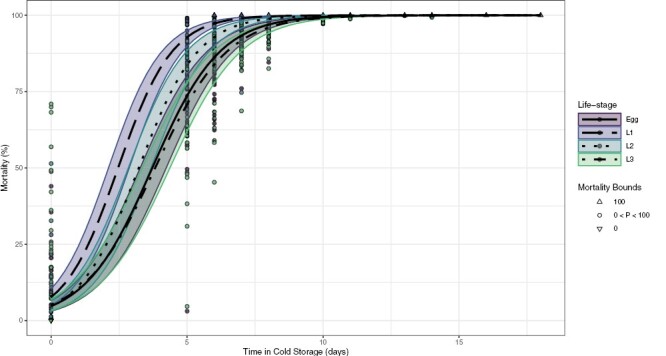
Mortality curves for 4 life stages of Mediterranean fruit fly (*Ceratitis capitata*), as predicted by the generalized linear mixed model (GLMM) fitted to the combined 2018 and 2019 data. Error bands around each line show the minimum to maximum predicted mortality, accounting for replicate, year, and cultivar effects. Points displayed are individual samples showing the estimated mortality, the shape representing probability boundaries (i.e., 0%, 100%, or in-between). Colors correspond to the life stage treated. L1 = first instars. L2 = second instars. L3 = third instars.

Results from the 2018 cold tolerance experiments investigating all 4 immature life stages showed that all life stages experienced 100% mortality after 10 days at 3 °C in most replicates, with only third instars (L3) in ‘Hayward’ kiwifruit having a median mortality less than 100% (Table 2). In ‘Zesy002’, third instars were the only life stage to have any survival after a 10-day treatment (Table 3). Modeling these data showed that third instars and eggs had similar mortality curves across all treatment durations (Fig. 4). When lethal time estimates were calculated, third instars were the most cold-tolerant of the 4 life stages tested, with an estimated LT_99.9968_ of 18.5 days (Table 4). Eggs were the second-most cold-tolerant life stage, with an LT_99.9968_ of 16.8 days.

**Table 2.** Summarized Mediterranean fruit fly (*Ceratitis capitata*) mortality results when reared in ‘Hayward’ kiwifruit and treated at 3 °C. L1 = first instars. L2 = second instars. L3 = third instars. D = Treatment duration (days). N = Number of boxes treated. P = Pupae retrieved. E = Estimated number treated over the 2 cold tolerance experiments. M = Median mortality (%). R = Mortality range (%)

	Eggs					L1					L2					L3				
D	N	P	E	M	R	N	P	E	M	R	N	P	E	M	R	N	P	E	M	R
0	12	2,892	3,082	2	0–35.5	12	2,420	2,560	2	0–25.8	12	2,371	2,473	0.7	0–17.2	32	10,039	10,964	0	0–70
5	12	973	2,898	68.8	3.1–88.1	12	81	2,427	97.8	91.6–99.1	12	399	2,376	84.3	71.4–93.5	12	1,176	2,469	59.3	4.6–82.2
6	12	575	2,898	79.8	65.9–97.8	12	16	2,427	99.5	97–100	12	292	2,376	88.4	78.1–96.8	12	483	2,469	83.4	58.3–98.3
7	12	232	2,898	91.7	83.8–100	12	3	2,427	100	99.1–100	12	78	2,360	97.2	92.5–98.3	12	242	2,404	91	81.9–95.4
8	12	121	2,898	96	89–100	12	1	2,427	100	99.5–100	12	12	2,376	99.5	98–100	32	382	10,044	96.4	82.6–99.8
10	12	2	2,898	100	99.6–100	12	0	2,427	100	100–100	12	2	2,376	100	99.4–100	31	55	9,491	99.5	97.1–100
11	–	–	–	–	–	–	–	–	–	–	–	–	–	–	–	20	16	7,575	100	98.8–100
13	–	–	–	–	–	–	–	–	–	–	–	–	–	–	–	19	1	7,244	100	99.8–100
14	–	–	–	–	–	–	–	–	–	–	–	–	–	–	–	20	3	7,575	100	99.7–100
16	–	–	–	–	–	–	–	–	–	–	–	–	–	–	–	20	0	7,575	100	100–100
18	–	–	–	–	–	–	–	–	–	–	–	–	–	–	–	20	0	7,575	100	100–100

**Table 3.** Summarized Mediterranean fruit fly (*Ceratitis capitata*) mortality results when reared in ‘Zesy002’ kiwifruit and treated at 3 °C. L1 = first instars. L2 = second instars. L3 = third instar. D = Treatment duration (days). N = Number of boxes treated. P = Pupae retrieved. E = Estimated number treated over the 2 cold tolerance experiments. M = Median mortality (%). R = Mortality range (%)

	Eggs					L1					L2					L3				
D	N	P	E	M	R	N	P	E	M	R	N	P	E	M	R	N	P	E	M	R
0	12	1,597	1,766	6.6	0–44	12	1,220	1,294	0	0–28.1	12	1,402	1,593	4.5	0–51.4	32	3,875	4,404	0	0–70.9
5	12	466	1,602	73.2	49.6–88.6	12	161	1,224	88.6	75.7–99.1	12	255	1,407	83.2	64.5–97	12	467	1,710	68.7	56.4–91.6
6	12	335	1,602	80.2	62.4–98.7	12	61	1,224	95.2	89.9–100	12	108	1,407	93.3	85–97	12	310	1,710	85.7	45.3–96.4
7	12	142	1,602	93.4	76–100	12	38	1,224	98.2	88.1–100	12	65	1,407	97.1	74.7–99	12	177	1,710	90.6	68.7–97.5
8	12	41	1,602	99.2	84.6–100	12	17	1,224	99	96.6–100	12	14	1,407	99.7	94.3–100	31	97	3,817	98.3	91.4–100
10	12	0	1,602	100	100–100	12	0	1,224	100	100–100	12	0	1,407	100	100–100	32	16	3,875	100	97.4–100
11	–	–	–	–	–	–	–	–	–	–	–	–	–	–	–	20	0	2,165	100	100–100
13	–	–	–	–	–	–	–	–	–	–	–	–	–	–	–	20	0	2,165	100	100–100
14	–	–	–	–	–	–	–	–	–	–	–	–	–	–	–	20	1	2,165	100	99.3–100
16	–	–	–	–	–	–	–	–	–	–	–	–	–	–	–	20	0	2,165	100	100–100
18	–	–	–	–	–	–	–	–	–	–	–	–	–	–	–	20	0	2,165	100	100–100

**Table 4.** Lethal time to 99.9968% mortality (LT_99.9968_, probit 9) estimates of required time in cold storage for each immature life stage of Mediterranean fruit fly (*Ceratitis capitata*) as predicted by the 2 generalized linear mixed models (GLMMs), along with associated 95% confidence intervals. L1 = first instars. L2 = second instars. L3 = third instars

Model data	Life stage	LT_99.9968_ estimate	95% CI
2018	Egg	16.8	15.0–19.3
2018 + 2019	Egg	16.4	15.1–18.1
2018	L1	12.7	11.4–14.5
2018 + 2019	L1	12.6	11.6–13.9
2018	L2	14.5	13.4–16.0
2018 + 2019	L2	14.3	13.4–15.3
2018	L3	18.5	16.6–21.4
2018 + 2019	L3	17.3	16.4–18.2

**Fig. 5.**
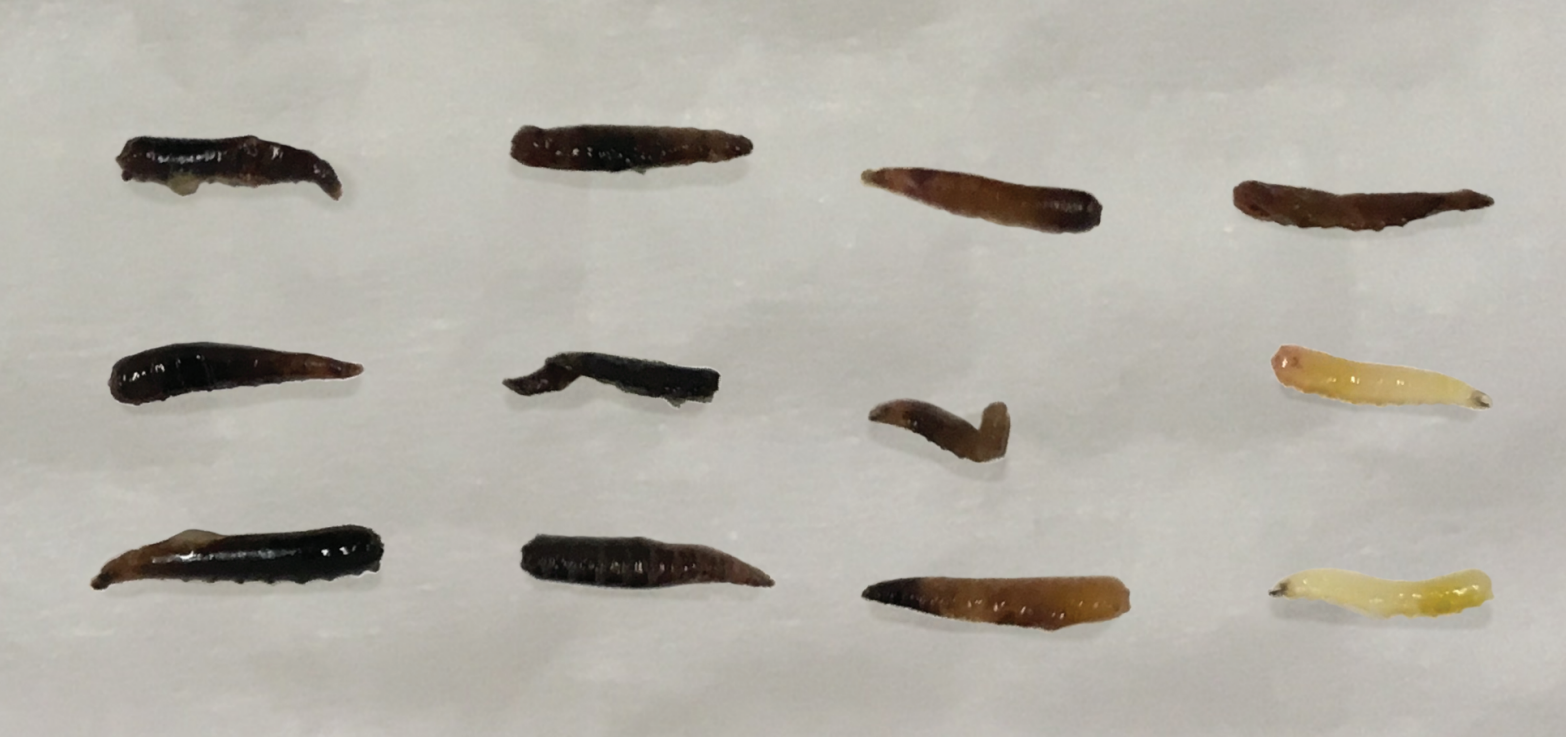
A sample of Mediterranean fruit fly (*Ceratitis capitata*) third instars found dead inside dissected fruit after treatment at 3 °C for 10 days, showing a high incidence of extensively melanized individuals.

During assessments, a high proportion of the dead larvae inside the fruit was found to be strongly melanized (Fig. 5), though exact numbers were not quantified. Affected specimens were often blackened through their whole body, though some were darker around the mouthparts or hindgut.

### Treatment Duration

Only third instars were subjected to longer treatment durations during the 2019 experiments. These showed a median mortality of 100% for all treatment times exceeding 10 days (Tables 2 and 3). Some replicates of treatment times exceeding 10 days yielded isolated survivors, but no surviving larvae were recovered from any replicate treated for 16 or 18 days (Tables 2 and 3).

Results from the combined 2018 + 2019 model showed no substantial changes to the lethality estimates relative to the 2018-only data. The 95% CI for this combined analysis was narrower (16.4–18.2 days compared with 16.6–21.4 days for the 2018-only data), reflecting the increased sampling of third instars (Table 4). Both analyses provided point estimates contained within the other analysis’ CIs (Table 4).

### Cultivar Differences

**Fig. 6.**
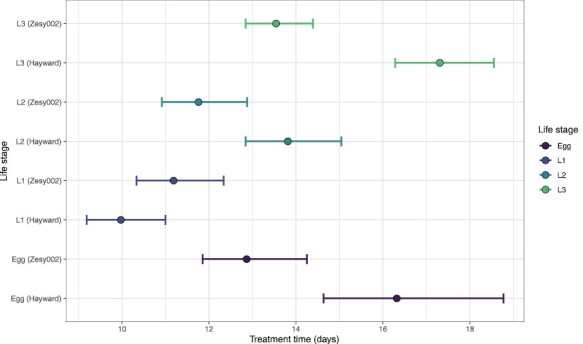
Estimates of the treatment duration at 3 °C required to achieve 99.9968% mortality (LT_99.9968_, probit 9) for 4 immature life stages of Mediterranean fruit fly (*Ceratitis capitata*) in ‘Zesy002’ and ‘Hayward’ kiwifruit, as estimated from models fitted to data across all years. Error bars show the associated 95% confidence intervals calculated using Fieller’s formula. Colors correspond to the life stages (L1 = first instars, L2 = second instars, L3 = third instars).

When kiwifruit cultivar was included as a fixed effect, it was shown to be significant (*P*-value ≤ 0.0001, *z*-value = 8.518). In both kiwifruit cultivars, third instars were the most cold tolerant (Fig. 6). Overall, flies reared in ‘Zesy002’ experienced higher average mortality than those in ‘Hayward’ (Table 5).

**Table 5.** Lethality estimates and 95% confidence intervals for Mediterranean fruit fly (*Ceratitis capitata*) immature life stages treated at 3 °C in 2 kiwifruit cultivars. LT_X_ = Estimated time in days to achieve X% mortality. L1 = first instars. L2 = second instars. L3 = third instars

Treated immature life stage	LT_50_	LT_95_	LT_99_	LT_99.9968_
**‘Zesy002’ kiwifruit**				
Egg	3.53 (2.81–4.09)	7.37 (6.95–7.85)	8.96 (8.43–9.65)	12.87 (11.86–14.25)
L1	2.92 (2.3–3.41)	6.32 (5.95–6.74)	7.73 (7.27–8.31)	11.19 (10.33–12.34)
L2	3.06 (2.44–3.55)	6.64 (6.28–7.04)	8.12 (7.67–8.68)	11.76 (10.92–12.88)
L3	3.42 (2.89–3.87)	7.58 (7.26–7.93)	9.31 (8.92–9.75)	13.54 (12.84–14.39)
**‘Hayward’ kiwifruit**				
Egg	3.85 (3.01–4.5)	7.4 (6.85–8.02)	9.39 (8.69–10.32)	16.32 (14.63–18.77)
L1	2.29 (1.73–2.76)	4.48 (4.08–4.87)	5.7 (5.29–6.16)	9.97 (9.19–11)
L2	3.33 (2.79–3.8)	6.32 (5.92–6.73)	7.99 (7.53–8.51)	13.81 (12.84–15.04)
L3	4.05 (3.44–4.59)	7.82 (7.38–8.27)	9.94 (9.45–10.48)	17.31 (16.28–18.56)

In all experiments where the 2 cultivars were infested, we found that *C. capitata* eggs inserted into ‘Zesy002’ kiwifruit had a suppressed hatch rate. This translated into greater numbers of pupae being consistently retrieved from ‘Hayward’ than ‘Zesy002’, with the control fruits yielding 10,147 and 7,515 pupae from ‘Hayward’ in 2018 and 2019, respectively, and 5,925 and 2,169 from ‘Zesy002’ despite the same number of each cultivar being infested at the same time.

### Temperature Profile of Treatments During Cold-Tolerance Experiments

The mean fruit core temperatures across all replicates in both years were between 2.69 and 3.54 °C (Table 6). Most replicates experienced a highly constant temperature profile, with 90% or more readings within 0.5 °C of the target temperature of 3 °C. Two replicates, located on the outside wall of their respective CS, had greater periods of time outside of this band. These undesirable temperatures only marginally exceeded 3.5 °C and did not have substantive effects on larval survival.

**Table 6.** Summary of the temperature profiles during the cold tolerance experiments testing all 4 Mediterranean fruit fly (*Ceratitis capitata*) immature life stages in 2018 and extended experiments testing third instars only in 2019. Replicate = 4 replicates were conducted in each of the 2 years of cold tolerance experiments. Date loaded = Date when fruit were moved into the cold store. Start = Mean fruit core temperatures recorded immediately after probes had been inserted. Overall mean = Mean temperature (°C) of all probes from fruit loading to fruit removal. Cool-down time = Mean duration (hours) from loading to when the fruit core probes read < 3.5 °C. Fruit core mean = Mean fruit core temperatures after the cool-down period until the end of the experiment. Standard deviation = Standard deviation of fruit core temperatures from the end of the cool-down period until the end of the experiment. Constancy % = Proportion of fruit core readings within ± 0.5 °C of 3 °C after the cool-down period

Replicate	Date loaded	Start (°C)	Overall mean (°C)	Cool-down time (h)	Fruit core mean (°C)	Standard deviation (°C)	Constancy %
1	21 Nov 2018	21.5	3.20	9.5	2.97	0.08	99.9
2	21 Nov 2018	24.3	3.68	17.3	3.32	0.13	96.8
3	23 Nov 2018	23.5	3.20	7.7	2.92	0.22	99.9
4	23 Nov 2018	24.8	3.76	81.4	3.54	0.24	42.3
1	15 Oct 2019	17.0	3.57	8.7	3.18	1.15	99.7
2	16 Oct 2019	19.1	3.59	6.3	2.69	0.14	89.3
3	17 Oct 2019	24.1	3.62	7.3	3.23	1.28	99.8
4	18 Oct 2019	23.2	4.00	12.3	3.51	0.22	56.6

### Large-Scale Trials

#### Ancillary trials.

The ancillary trial, which counted the number of pupae from each of 30 infested ‘Hayward’ kiwifruit, had a mean of 51 pupae emerging from each fruit (standard error = 0.987, 95% CI = 48.9–53.0 pupae per fruit).

#### Dissections.

Dissections showed that third instars were present in high proportions, exceeding 85% of the total number of larvae in each fruit (Table 7). The mean number of live third instars in each fruit was 77.1 in Replicate 1 (95% CI = 74.1–80.1), 74.3 in Replicate 2 (95% CI = 71.1–77.5), and 77.6 in Replicate 3 (95% CI = 75.0–80.1).

**Table 7.** Total numbers of each Mediterranean fruit fly (*Ceratitis capitata*) immature life stage recovered during dissections of 10 ‘Hayward’ kiwifruit to confirm the treated life stage in large-scale trials. L1 = first instar, L2 = second instar, L3 = third instar, % = percentage of third instars, Mean = mean number of living third instars found in each fruit, SE = standard error of the mean number of live third instars in each fruit

Replicate	Eggs	L1	L2	L3	%	Mean live L3/fruit	SE
1	0	27	62	804	90	77.1	1.53
2	0	33	78	772	87.4	74.3	1.65
3	0	33	63	790	89.3	77.6	1.28

#### Pupal emergence from experimental controls.

Large numbers of pupae emerged from the 200 control fruits infested at the same time as treated fruits. A total of 10,226 pupae were counted in Replicate 1, Replicate 2 yielded 10,598 pupae, while Replicate 3 had 10,625 pupae recovered (Table 8). The mean number of larvae per 200 fruits was 10,483 (standard error = 128, 95% CI = 9,929–11,036).

**Table 8.** Number of pupae recovered during the large-scale trials from fruit infested with Mediterranean fruit fly (*Ceratitis capitata*) third instars after treatment at 3°C

Days treated	Number of fruit per replicate	Replicate 1	Replicate 2	Replicate 3
0	200	10,226	10,598	10,625
8	180	187	188	188
10	180	41	37	40
18	3,400	0	0	0

#### Pupal emergence from treated fruit.

We recovered 563 pupae from the 3 replicates of infested fruit treated for 8 days (Table 8). This decreased to 118 pupae recovered from the 3 replicates of fruit treated for 10 days (Table 8). No pupae were recovered from any of the 3,400 fruits treated at 3 °C for 18 days in any of the 3 replicates of the experiment (Table 8).

#### Estimates of the number of insects treated.

Three sources of evidence were used for estimating the number of insects treated during the large-scale trial. These were: (i) the number of pupae that emerged during ancillary trials, (ii) the number of larvae recovered during dissections at the start of the treatments, and (iii) the number of pupae recovered from the control fruit infested in the experiment.

#### Extrapolation from ancillary trials.

Extrapolating from the number of pupae that emerged from the ancillary trial fruit, we calculated that between 9,796 and 10,603 pupae would emerge from the 200 control fruits in the large-scale trials. The accuracy of this prediction is demonstrated by the number of pupae observed in each of the 3 control replicates falling within this interval (Table 8), giving confidence that this calculation accurately estimates the number of larvae killed during the 18-day treatment.

Using this method, we calculate that the 3,400 kiwifruit infested in each replicate could be reasonably expected to have yielded between 166,534 and 180,265 *C. capitata* pupae per replicate, with a total of between 499,604 and 540,796 pupae across all replicates, had these fruits not been treated.

#### Extrapolation from larval numbers in dissections.

Extrapolating from the number of third instars found during dissections, we calculate that Replicate 1 treated between 251,938 and 272,341 third instars; Replicate 2 treated between 241,600 and 263,639 larvae; and Replicate 3 between 255,282 and 272,397 larvae. This corresponds to between 748,820 and 808,377 larvae estimated to have been treated across all 3 replicates.

#### Extrapolation from experimental controls.

Extrapolating from the number of pupae counted from the control fruits from the 3 replicates, we calculate that each replicate of 3,400 infested kiwifruit would have yielded between 168,794 and 187,627 pupae had they not been treated. Across all 3 replicates, we calculate that between 506,383 and 562,882 pupae could have been reasonably expected to have emerged from our treated fruit without the 18-day treatment. Alternative methods to estimate this number arrive at 515,461 individuals treated when using Equation 3 of Wright et al. (2023) or 515,926 individuals treated when using the formula provided by the IPPC (2023, p 113).

Using Formula 3 of Couey and Chew (1986), we calculate that these results indicate that we have greater than 99% confidence that the actual mortality of third-instar *C. capitata* in kiwifruit induced by our 18-day treatment at 3 °C exceeded 99.9968%.

### Temperature Profiles

Temperatures were stable throughout the duration of the large-scale treatments. At the time that probes were inserted, the fruit core temperatures were about 25 °C. After loading, it took between 48 and 55 h for fruit core temperatures to drop below 3.5 °C (Table 9). These correspond to a cool-down rate of about 0.009 °C min^−1^. Once reached, the fruit core temperatures stayed consistently within ± 0.5 °C of the target temperature of 3 °C, with a mean temperature of 2.8 °C across all 3 replicates.

**Table 9.** Summary of the temperature profiles during the large-scale trials testing a phytosanitary treatment protocol on Mediterranean fruit fly (*Ceratitis capitata*) third-instar larvae. Date loaded = Date when fruit were moved into the cold store. Start = Mean fruit core temperatures recorded immediately after probes had been inserted. Overall mean = Mean temperature (°C) of all probes from fruit loading to fruit removal. Cool-down time = Duration (hours) from loading to when 6 fruit core probes read < 3.5 °C. Fruit core mean = Mean fruit core temperatures from the time at least 6 probes read < 3.5 °C until the end of the experiment. Standard deviation = Standard deviation of fruit core temperatures from the end of the cool-down period until the end of the experiment. Constancy % = Proportion of fruit core readings within ± 0.5 °C of 3 °C after the cool-down period

Replicate	Date loaded	Start (°C)	Overall mean (°C)	Cool-down time (h)	Fruit core mean (°C)	Standard deviation (°C)	Constancy %
1	11 Jan 2022	24.8	3.53	54.2	3.04	0.48	79.3
2	12 Jan 2022	24.1	3.50	49.7	2.93	0.54	62.3
3	13 Jan 2022	25.2	3.50	48.6	3.05	0.87	66.3

## Discussion

Our results demonstrate that an 18-day, 3 °C cold treatment is an effective phytosanitary disinfestation protocol for *C. capitata* in ‘Hayward’ and ‘Zesy002’ kiwifruit. They are consistent with other results that found that no *C. capitata* emerged from a mixture of infested fruits, including apples, peaches, and kamani nuts, after 16 days of being treated at 36–40°F (i.e., 2.2–4.4 °C) (Back and Pemberton 1916). Our proposed cold treatment is the same protocol as has been accepted by the IPPC as a suitable treatment for *C. capitata* in lemons (IPPC 2017b) and slightly shorter than other proposed treatments for *C. capitata* in other commodities, such as 20 days at 3 °C for flies in *Citrus sinensis* and *Citrus reticulata* (De Lima et al. 2007, IPPC 2017c, a) and in table grapes (*Vitis vinifera*) (De Lima et al. 2011).

The influence that commodities have on fruit fly tolerance to cold treatment remains a vexing issue. Indeed, there are statistically discernible differences between different fruits and cultivars. For example, the LT_95_ values reported for all life stages in 5 citrus varieties treated at 3 °C were higher than what we found in either kiwifruit cultivar (De Lima et al. 2007). At lower mortality levels, however, there were overlaps in the reported LT_50_ values of egg and third-instar larvae of *C. capitata* treated in citrus (De Lima et al. 2007) with our estimates of cold tolerance in ‘Hayward’ kiwifruit, though mortality estimates in citrus remain higher across all life stages than what we estimated for ‘Zesy002’ kiwifruit. Similarly, our data on the cold tolerance of *C. capitata* eggs and third instars treated in ‘Hayward’ overlap with the reported LT_50_ and LT_99_ values of *C. capitata* treated in 3 grape cultivars at 3 °C (De Lima et al. 2011), but they estimated significantly higher values of these parameters for first and second instars than shown by our data. In addition, we estimated a lower cold tolerance of *C. capitata* in ‘Zesy002’ kiwifruit than in any of the grape cultivars. However, this statistical significance is strongly driven by differences between fruits and cultivars in the intermediate treatment durations, which are irrelevant for phytosanitary treatments. The magnitude of these differences decreases with treatment time, and at the durations of interest for phytosanitary treatments, the effect size between various fruits and cultivars becomes relatively small. The lack of differences among treatments at these durations suggests that an 18-day 3 °C cold treatment is likely a reasonable starting point for phytosanitary treatments of *C. capitata* in other kiwifruit cultivars for which we do not yet have data. The New Zealand National Plant Protection Organization, the Ministry of Primary Industries, came to a similar conclusion in their assessment of the approved phytosanitary cold treatments for Tephritidae on citrus (Urlacher and Devitt 2021).

Our results also demonstrate that the results from our 2018 cold tolerance experiments accurately predicted that an 18-day treatment at 3 °C would be a suitable protocol. If cold tolerance experiments on additional kiwifruit cultivars conducted at a similar scale yield comparable results, it would be reasonable to expect that an 18-day 3 °C cold treatment would likewise be effective for these cultivars without requiring further, resource-intensive, large-scale trials to be carried out.

Our results are also consistent with several other studies on *C. capitata* indicating that the third instar is the most cold-tolerant life stage (Back and Pemberton 1916, Hashem et al. 2004, Gazit et al. 2014, Ware and Toit 2017, Hallman et al. 2019). One notable exception is the work by De Lima (1992) on ‘Hayward’ kiwifruit, which concluded that the first instar was more tolerant than the third. It is unclear what might explain this discrepancy, though variable rates of larval development caused by differences in fruit maturity, pH, or soluble solids content might be involved. Anecdotal indications from our research suggest that *C. capitata* larvae develop more uniformly and with lower mortality in more mature kiwifruit. De Lima (1992) does not discuss the maturity of the fruit used in his experiments, making it difficult to gauge the likelihood of this factor influencing the outcomes of these different experiments. However, his overall conclusions, demonstrating that 12 days at 0 °C or 14 days at 1 °C was sufficient for total mortality of fruit fly larvae in trials using over 13,000 individuals (De Lima 1992), do not disagree with our present results.

Although the standard storage temperature of kiwifruit is 0 °C (Burdon and Lallu 2011), these experiments focused on a treatment temperature of 3 °C. This elevated temperature relative to the industry target will allow disinfestation protocols based on these data to be readily implemented as part of a standard industry cold chain and be robust to the presence of temperature spikes that are inevitably present during transit.

Several comparative studies have shown that *C. capitata* is one of the more cold-tolerant species of the Dacinae subfamily, which contains many of the Tephritidae species of economic importance (Jessup et al. 1993, Armstrong et al. 1995, Hashem et al. 2004, De Lima et al. 2007, 2011, Hallman et al. 2013). Comparative research into *C. capitata*, *Zeugodacus cucurbitae* (Coquillett) (melon fly), and *Bactrocera dorsalis* (Hendel) (Oriental fruit fly) showed that *C. capitata* was more likely to survive treatments at 1 °C for 7 days or longer than either of the other 2 species (Armstrong et al. 1995). These comparative experiments did not include the cucurbit-favoring *Z. tau* (Walker), which seems to be very tolerant of cold temperatures, as demonstrated by small numbers of third instars remaining alive after 20 days at 1.4 °C (Dias et al. 2023), though no adults emerged from larvae that had been treated for more than 13 days (Dias et al. 2023). Experiments have also demonstrated that *C. capitata* requires longer treatment times at 2 and 3 °C than *B. tryoni* in citrus fruits (De Lima et al. 2007). The relative persistence of *C. capitata* can be inferred to hold true in kiwifruit also, with *B. tryoni* unable to tolerate treatment times greater than 12 days at 2.5 °C in ‘Zesy002’ (or Gold3, an alternate name used by the authors) kiwifruit (Balagawi et al. 2021). The disinfestation protocol we recommend here will likely be effective against most of the economically important Dacinae that concern the kiwifruit industry; however, applying this disinfestation schedule to members of other Tephritidae subfamilies may be more problematic. Although species of *Anastrepha*, including *Anastrepha fraterculus* Wiedemann (South American fruit fly), show a similar cold tolerance to *C. capitata* (Willink et al. 2006, Hallman et al. 2017, Dias et al. 2020) and might be adequately controlled with the protocol demonstrated here, other species within the Trypetinae are known to be substantially more tolerant of cold temperatures. Although a cold treatment protocol is available for the larvae of *Rhagoletis pomonella* (Walsh) (apple maggot) (Government of Canada 2020), the long duration of the treatment (0.6 °C (33 °F) for 42 days or 3.3 °C (38 °F) for 90 days), has inspired the search for alternative treatments (Agnello et al. 2002, Hallman 2004, Hulasare et al. 2013). In addition, diapausing pupae of *R. pomonella* and *Rhagoletis indifferens* Curran (Western cherry fruit fly) can survive several weeks of exposure to temperatures as low as −10 °C (Aliniazee 1975, Yee et al. 2015). A 3 °C cold treatment of 18 days duration would be inadequate for these or related species.

The observation of strongly melanized dead larvae during assessments suggests potential mechanisms for the lethality of extended cold temperatures. Melanin is a key component of insect immunity function (González-Santoyo and Córdoba-Aguilar 2012), with roles in encapsulating pathogens and wound healing (Gillespie et al. 1997, Krautz et al. 2014). Melanization processes are localized to the site of the injury and can form rapidly after damage has been sustained (Galko and Krasnow 2004, Tang 2009, González-Santoyo and Córdoba-Aguilar 2012). Observations in these experiments suggest that *C. capitata* larvae held for prolonged durations at 3 °C sustain extensive tissue wounding. Specimens that show darker regions around the hindgut suggest that wounding of the gut, leading to microbial infiltration of the hemocoel and/or disrupted osmotic regulation, may be occurring. This hypothesized mechanism is consistent with results from experiments of cold tolerance in crickets, which indicate that injuries to the gut are linked with lethal responses to cold temperatures (MacMillan and Sinclair 2011).

The cool-down times in these experiments were shorter than commercial kiwifruit cooling regimes, which take about 5 days for the fruit to reach 2 °C (Burdon and Lallu 2011). Insects can respond to their changing environment through various physiological mechanisms, increasing their resistance to cold damage (Teets and Denlinger 2013, Overgaard and MacMillan 2017). These cold hardening responses can be induced by exposure to a static conditioning temperature (Koveos 2001, Nyamukondiwa et al. 2010) or by subjecting insects to a slow cooling rate (Meats 1987, Bale et al. 1989, Koveos 2001). Rapid physiological changes may allow the induction of cold hardening during cool-down periods as part of fruit treatments. *Ceratitis capitata* adults have been shown to display this rapid cold hardening response, with experiments demonstrating that a 2-h exposure to temperatures of 5 °C substantially increases survival after a 2-h exposure to −5 °C (Nyamukondiwa et al. 2010). Cold hardening persisted for over 8 h after exposure to the hardening temperatures (Nyamukondiwa et al. 2010). However, the cool-down rates observed in these experiments were between 0.05 and 0.004 °C min^−1^, an order of magnitude slower than the cooling rates examined in physiological experiments. These slow cool-down rates suggest that the experiments reported here likely have already covered the risk of increased longevity of cold-hardened larvae. In addition, physiological research on rapid cold hardening has looked primarily at their impact on acute mortality, with lethal temperatures maintained for only a few hours during challenges. The extent to which this induced cold hardening protects insects from the chronic effects of extended chilling periods has yet to be examined by insect physiologists. However, the success of cold-temperature phytosanitary treatments against tephritid fruit flies suggests that these cold hardening processes are insufficient to cause issues, given sufficiently long treatment times. Understanding the physiological processes involved in inducing mortality and the ability of insects to combat and recover from this damage will help identify the potential consequences of temperature spikes during commercial treatments.

The results presented here demonstrate that cold treatments of 3.5 °C or below for 18 days or longer effectively kill third instar and younger *C. capitata* larvae in ‘Hayward’ and ‘Zesy002’ kiwifruit, with a mortality rate that exceeds 99.9968% (probit 9). The number of larvae treated in large-scale trials allows us to infer this result with a greater than 99% degree of confidence. These results support a disinfestation protocol that can be readily integrated into standard kiwifruit production and export practices.

## References

[CIT0001] Agnello AM , Spangler SM, Minson ES, Harris T, Kain DP. Effect of high-carbon dioxide atmospheres on infestations of apple maggot (Diptera: Tephritidae) in apples. J Econ Entomol. 2002:95(2):520–526. 10.1603/0022-0493-95.2.520 12020036

[CIT0002] Aitken AG , Warrington IJ. Fresh facts. New Zealand horticulture 2020. Auckland: Horticulture New Zealand and Plant & Food Research; 2020.

[CIT0003] Aitken AG , Warrington IJ. Fresh facts. New Zealand horticulture 2021 . Auckland: Horticulture New Zealand and Plant & Food Research; 2021.

[CIT0004] Aliniazee MT. Susceptibility of diapausing pupae of the western cherry fruit fly (Diptera: Tephritidae) and a parasite (Hymenoptera: Diapriidae) to subfreezing temperatures. Environ Entomol. 1975:4(6):1011–1013. 10.1093/ee/4.6.1011

[CIT0005] Arita LH. Reproductive and sexual maturity of the Mediterranean fruit fly, *Ceratitis capitata* (Wiedemann). Proc Hawaii Entomol Soc. 1982:24(1):25–29.

[CIT0006] Armstrong JW , Silva ST, Shishido VM. Quarantine cold treatment for Hawaiian carambola fruit infested with Mediterranean fruit fly, melon fly, or oriental fruit fly (Diptera: Tephritidae) eggs and larvae. J Econ Entomol. 1995:88(3):683–687. 10.1093/jee/88.3.683

[CIT0007] ASTM E562-11. Standard practice for preparation and use of an ice-point bath as a reference temperature. Book of Standards. 2016:14(03):1–4. 10.1520/E0563-11R16

[CIT0008] Back EA , Pemberton CE. Effect of cold-storage temperatures upon the Mediterranean fruit fly. J Agric Res. 1916:5(15):657–666.

[CIT0009] Balagawi S , Archer J, Cruickshank D, Cruickshank C, Barchia I. Cold treatment: an effective post-harvest disinfestation treatment for *Bactrocera tryoni* (Diptera: Tephritidae) in ‘gold3’ kiwifruit. Austral Entomol. 2021:60(3):621–627. 10.1111/aen.12561.

[CIT0010] Bale JS , Hansen TN, Nishino M, Baust JG. Effect of cooling rate on the survival of larvae, pupariation, and adult emergence of the gallfly *Eurosta solidaginis*. Cryobiology. 1989:26(3):285–289. 10.1016/0011-2240(89)90024-2 2743790

[CIT0011] Bateman MA. The ecology of fruit flies. Annu Rev Entomol. 1972:17(1):493–518. 10.1146/annurev.en.17.010172.002425

[CIT0012] Bonizzoni M , Guglielmino CR, Smallridge CJ, Gomulski M, Malacrida AR, Gasperi G. On the origins of medfly invasion and expansion in Australia. Mol Ecol. 2004:13(12):3845–3855. 10.1111/j.1365-294X.2004.02371.x 15548296

[CIT0013] Brooks ME , Kristensen K, Van Bentham KJ, Magnusson A, Berg CW, Nielsen A, Skaug HJ, Machler M, Bolker BM. glmmTMB balances speed and flexibility among packages for zero-inflated generalized linear mixed modeling. The R Journal 2017:9(2):378–400. 10.3929/ethz-b-000240890

[CIT0014] Burdon J , Lallu N. 14—Kiwifruit (*Actinidia* spp.). In: Yahia EM editor. Postharvest biology and technology of tropical and subtropical fruits. Cambridge (UK): Woodhead Publishing; 2011. p. 326–362e. 10.1533/9780857092885.326

[CIT0015] Cockayne AH. On the occurrence of *Ceratitis capitata* [the Mediterranean fruit-fly] in New Zealand. Trans Proc New Zealand Inst 1907:40:564.

[CIT0016] Couey HM , Chew V. Confidence limits and sample size in quarantine research. J Econ Entomol. 1986:79(4):887–890. 10.1093/jee/79.4.887

[CIT0017] De Lima CPF. Disinfestation of kiwifruit using cold-storage as a quarantine treatment for Mediterranean fruit fly (*Ceratitis capitata* Wiedemann). NZ J Crop Hortic Sci. 1992:20(2):223–227. 10.1080/01140671.1992.10421919

[CIT0018] De Lima CPF , Jessup AJ, Cruickshank L, Walsh CJ, Mansfield ER. Cold disinfestation of citrus (*Citrus* spp.) for Mediterranean fruit fly (*Ceratitis capitata*) and Queensland fruit fly (*Bactrocera tryoni*) (Diptera: Tephritidae). NZ J Crop Hortic Sci. 2007:35(1):39–50. 10.1080/01140670709510166

[CIT0019] De Lima CPF , Jessup AJ, Mansfield ER, Daniels D. Cold treatment of table grapes infested with Mediterranean fruit fly *Ceratitis capitata* (Wiedemann) and Queensland fruit fly *Bactrocera tryoni* (Froggatt) Diptera: Tephritidae. NZ J Crop Hortic Sci. 2011:39(2):95–105. 10.1080/01140671.2010.526620

[CIT0020] De Lima CPF , Mansfield ER, Poogoda SR. International market access for Australian tablegrapes through cold treatment of fruit flies with a review of methods, models and data for fresh fruit disinfestation. Aust J Grape Wine Res. 2017:23(3):306–317. 10.1111/ajgw.12284

[CIT0021] De Meyer M , Copeland RS, Wharton RA, McPheron BA. On the geographic origin of the Medfly *Ceratitis capitata* (Wiedemann) (Diptera: Tephritidae). In: Barnes BN, editor. Proceedings of 6th International Fruit Fly Symposium, 6-10 May 2002, Stellenbosch, South Africa. Irene (South Africa): Isteg Scientific Publications; 2002. p. 45–53.

[CIT0022] Dias VS , Hallman GJ, Araújo AS, Lima IVG, Galvão-Silva FL, Caravantes LA, Rivera MNG, Aguilar JS, Cáceres-Barrios CE, Vreysen MJB, et al. High cold tolerance and differential population response of third instars from the *Zeugodacus tau* complex to phytosanitary cold treatment in navel oranges. Postharvest Biol Technol. 2023:203:112392. 10.1016/j.postharvbio.2023.112392

[CIT0023] Dias VS , Hallman GJ, Cardoso AAS, Hurtado NV, Rivera C, Maxwell F, Cáceres-Barrios CE, Vreysen MJB, Myers SW. Relative tolerance of three morphotypes of the *Anastrepha fraterculus* complex (Diptera: Tephritidae) to cold phytosanitary treatment. J Econ Entomol. 2020:113(3):1176–1182. 10.1093/jee/toaa027 32161970 PMC7275689

[CIT0024] Ferguson AR. Chapter two—Kiwifruit: the wild and the cultivated plants. In: Boland M and Moughan PJ, editors. Advances in food and nutrition research, vol. 68. Waltham (MA): Academic Press; 2013. p. 15–32. 10.1016/B978-0-12-394294-4.00002-X 23394980

[CIT0025] Fieller EC. Some problems in interval estimation. J R Stat Soc Ser B Methodol 1954:16(2):175–185. 10.1111/j.2517-6161.1954.tb00159.x

[CIT0026] Fletcher BS. The biology of dacine fruit flies. Annu Rev Entomol. 1987:32(1):115–144. 10.1146/annurev.en.32.010187.000555

[CIT0027] Galko MJ , Krasnow MA. Cellular and genetic analysis of wound healing in *Drosophila* larvae. PLoS Biol. 2004:2(8):E239. 10.1371/journal.pbio.0020239 15269788 PMC479041

[CIT0028] Gazit Y , Akiva R, Gavriel S. Cold tolerance of the Mediterranean fruit fly in date and mandarin. J Econ Entomol. 2014:107(5):1745–1750. 10.1603/EC14050 26309262

[CIT0029] Gillespie JP , Kanost MR, Trenczek T. Biological mediators of insect immunity. Annu Rev Entomol. 1997:42(1):611–643. 10.1146/annurev.ento.42.1.611 9017902

[CIT0030] González-Santoyo I , Córdoba-Aguilar A. Phenoloxidase: a key component of the insect immune system. Entomol Exp Appl. 2012:142(1):1–16. 10.1111/j.1570-7458.2011.01187.x

[CIT0031] Government of Canada. D-00-07: Phytosanitary requirements to prevent the introduction and spread of apple maggot, *Rhagoletis pomonella* (Walsh). 10th revision. Ottawa: Canadian Food Inspection Agency; 2020. https://inspection.canada.ca/plant-health/invasive-species/directives/horticulture/d-00-07/eng/1323819375916/1323819810662

[CIT0032] Hallman G , Wang L, Uzel GD, Cancio-Martinez E, Cáceres-Barrios CE, Myers SW, Vreysen MJB. Comparison of populations of *Ceratitis capitata* (Diptera: Tephritidae) from three continents for susceptibility to cold phytosanitary treatment and implications for generic cold treatments. J Econ Entomol. 2019:112(1):127–133. 10.1093/jee/toy331 30346545

[CIT0033] Hallman GJ. Irradiation disinfestation of apple maggot (Diptera: Tephritidae) in hypoxic and low-temperature storage. J Econ Entomol. 2004:97(4):1245–1248. 10.1603/0022-0493-97.4.1245 15384333

[CIT0034] Hallman GJ , Maset BA, Martínez EIC, Cáceres Barrios CE, Vreysen MJB, Myers SW, Wornoayporn V. Phytosanitary cold treatment against *Anastrepha grandis* (Diptera: Tephritidae). Fla Entomol. 2017:100(1):29–31. 10.1653/024.100.0106.

[CIT0035] Hallman GJ , Myers SW, El-Wakkad MF, Tadrous MD, Jessup AJ. Development of phytosanitary cold treatments for oranges infested with *Bactrocera invadens* and *Bactrocera zonata* (Diptera: Tephritidae) by comparison with existing cold treatment schedules for *Ceratitis capitata* (Diptera: Tephritidae). J Econ Entomol. 2013:106(4):1608–1612. 10.1603/ec13066 24020272

[CIT0036] Hartig F. DHARMa: residual diagnostics for hierarchical (multi-level/mixed) regression models. Version 0.4.6; 2020 [accessed 2023 November 29]. https://CRAN.R-project.org/package=DHARMa

[CIT0037] Hashem AG , Soliman NA, Soliman AM. Effect of low temperatures on eggs and larvae of Mediterranean fruit fly and peach fruit fly inside fruits as a quarantine procedure. Ann Agric Sci Moshtohor 2004:42(1):345–356.

[CIT0038] Holder PW , Stephenson B, Chadfield K, Frampton R. The finding of Mediterranean fruit fly in Auckland, New Zealand and the Ministry of Agriculture’s response. The Weta 1997:20:4–6.

[CIT0039] Hulasare R , Payton ME, Hallman GJ, Phillips TW. Potential for hypobaric storage as a phytosanitary treatment: mortality of *Rhagoletis pomonella* (Diptera: Tephritidae) in apples and effects on fruit quality. J Econ Entomol. 2013:106(3):1173–1178. 10.1603/ec12343 23865181

[CIT0040] IPPC. International standards for phytosanitary measures. ISPM 28 Phytosanitary treatments for regulated pests. PT 24: cold treatment for *Ceratitis capitata* on *Citrus sinensis*. Rome: Secretariat of the International Plant Protection Convention; 2017a. https://www.ippc.int/en/publications/84350/

[CIT0041] IPPC. International standards for phytosanitary measures. ISPM 28 phytosanitary treatments for regulated pests. PT 25: cold treatment for *Ceratitis capitata* on *Citrus reticulata* × *C. sinensis*. Rome: Secretariat of the International Plant Protection Convention; 2017c. https://www.ippc.int/en/publications/84351/

[CIT0042] IPPC. International standards for phytosanitary measures. ISPM 28 phytosanitary treatments for regulated pests. PT 26: cold treatment for *Ceratitis capitata* on *Citrus limon*. Rome: Secretariat of the International Plant Protection Convention; 2017b. https://www.ippc.int/en/publications/84352/

[CIT0043] IPPC. IPPC Procedure Manual for Standard Setting (2022–2023). Rome: International Plant Protection Convention; 2023. p. 193. https://www.ippc.int/en/publications/85024/

[CIT0044] Jessup AJ , De Lima CPF, Hood CW, Sloggett RF, Harris AM, Beckingham M. Quarantine disinfestation of lemons against *Bactrocera tryoni* and *Ceratitis capitata* (Diptera: Tephritidae) using cold storage. J Econ Entomol. 1993:86(3):798–802. 10.1093/jee/86.3.798

[CIT0045] Karsten M , Jansen van Vuuren B, Addison P, Terblanche JS. Deconstructing intercontinental invasion pathway hypotheses of the Mediterranean fruit fly (*Ceratitis capitata*) using a Bayesian inference approach: are port interceptions and quarantine protocols successfully preventing new invasions? Divers Distrib. 2015:21(7):813–825. 10.1111/ddi.12333

[CIT0046] Koveos DS. Rapid cold hardening in the olive fruit fly *Bactrocera oleae* under laboratory and field conditions. Entomol Exp Appl. 2001:101(3):257–263. 10.1046/j.1570-7458.2001.00910.x

[CIT0047] Krautz R , Arefin B, Theopold U. Damage signals in the insect immune response. Front Plant Sci. 2014:5(342):1–11. 10.3389/fpls.2014.00342 PMC4093659 25071815

[CIT0048] Liquido NJ , Cunningham RT, Nakagawa S. Host plants of Mediterranean fruit fly (Diptera: Tephritidae) on the Island of Hawaii (1949-1985 Survey). J Econ Entomol. 1990:83(5):1863–1878. 10.1093/jee/83.5.1863

[CIT0049] MacMillan HA , Sinclair BJ. The role of the gut in insect chilling injury: cold-induced disruption of osmoregulation in the fall field cricket, *Gryllus pennsylvanicus*. J Exp Biol. 2011:214(Pt 5):726–734. 10.1242/jeb.051540 21307058

[CIT0050] Meats A. Survival of step and ramp changes of temperature by the Queensland fruit-fly, *Dacus tryoni*. Physiol Entomol. 1987:12(2):165–170. 10.1111/j.1365-3032.1987.tb00737.x

[CIT0051] Myers SW , Cancio-Martinez E, Hallman GJ, Fontenot EA, Vreysen MJB. Relative tolerance of six *Bactrocera* (Diptera: Tephritidae) species to phytosanitary cold treatment. J Econ Entomol. 2016:109(6):2341–2347. 10.1093/jee/tow206 27660425

[CIT0052] Nyamukondiwa C , Kleynhans E, Terblanche JS. Phenotypic plasticity of thermal tolerance contributes to the invasion potential of Mediterranean fruit flies (*Ceratitis capitata*). Ecol Entomol. 2010:35(5):565–575. 10.1111/j.1365-2311.2010.01215.x

[CIT0053] Overgaard J , MacMillan HA. The integrative physiology of insect chill tolerance. Annu Rev Physiol. 2017:79:187–208. 10.1146/annurev-physiol-022516-034142 27860831

[CIT0054] Papadopoulos NT , Katsoyannos BI, Carey JR, Kouloussis NA. Seasonal and annual occurrence of the Mediterranean fruit fly (Diptera: Tephritidae) in Northern Greece. Ann Entomol Soc Am. 2001:94(1):41–50. 10.1603/0013-8746(2001)094[0041:saaoot]2.0.co;2

[CIT0055] Pather V , MacLellan R, King K. National fruit fly surveillance programme annual report. Surveillance 2019:46(3):83–86.

[CIT0056] R Core Team. R: A language and environment for statistical computing. Vienna (Austria): R Foundation for Statistical Computing; 2023.

[CIT0057] Ricalde MP , Nava DE, Loeck AE, Donatti MG. Temperature-dependent development and survival of Brazilian populations of the Mediterranean fruit fly, *Ceratitis capitata*, from tropical, subtropical and temperate regions. J Insect Sci. 2012:12(1):33. 10.1673/031.012.3301 22963468 PMC3471798

[CIT0058] Ruiz-Arce R , Todd TN, Deleon R, Barr NB, Virgilio M, De Meyer M, McPheron BA. Worldwide phylogeography of *Ceratitis capitata* (Diptera: Tephritidae) using mitochondrial DNA. J Econ Entomol. 2020:113(3):1455–1470. 10.1093/jee/toaa024 32112108

[CIT0059] Segura DF , Vera MT, Cladera JL. Host utilization by the Mediterranean fruit fly, *Ceratitis capitata* (Diptera: Tephritidae). In: Barnes BN, editor. Proceedings of the 6th International Fruit Fly Symposium, 6–10 May 2002, Stellenbosch, South Africa. Irene (South Africa): Isteg Scientific Publications; 2002. p. 83–90.

[CIT0060] Shoukry, AM, Hafez M. Studies on the biology of the Mediterranean fruit fly *Ceratitis capitata*. Entomol Exp Appl. Hafez 1979:26(1):33–39. 10.1111/j.1570-7458.1979.tb02894.x.

[CIT0061] Tang H. Regulation and function of the melanization reaction in *Drosophila*. Fly 2009:3(1):105–111. 10.4161/fly.3.1.7747 19164947

[CIT0062] Teets NM , Denlinger DL. Physiological mechanisms of seasonal and rapid cold-hardening in insects. Physiol Entomol. 2013:38(2):105–116. 10.1111/phen.12019

[CIT0063] Urlacher E , Devitt J. A novel method of determining a cold treatment for fruit flies associated with citrus, MPI Technical paper 2021/31. Wellington: Ministry for Primary Industries Manatū Ahu Matua; 2021. p. 25.

[CIT0064] Ware AB , Toit CLND. Cold disinfestation of ‘Hass’ avocado (*Persia americana*) of three species of fruit fly (Diptera: Tephritidae)—*Ceratitis capitata*, *Ceratitis rosa*, and *Ceratitis cosyra*. J Econ Entomol. 2017:110(3):954–960. 10.1093/jee/tox068 28444314

[CIT0065] Wickham, H. stringr: Simple, consistent wrappers for common string operations. Version 1.5.0; 2022 [accessed 2023 November 29]. https://CRAN.R-project.org/package=stringr

[CIT0066] Willink E , Gastaminza G, Salvatore A, Gramajo MC, Acenolaza M, Avila R, Favre P. Quarantine cold treatments for *Ceratitis capitata* and *Anastrepha fraterculus* (Diptera: Tephritidae) for citrus in Argentina: conclusions after 10 years of research. In: Sugayam R, Zucchi RA, Ovruski SM, Sivinski J, editors. Fruit flies of economic importance: from basic to applied knowledge. Proceedings of the 7th International Symposium on Fruit Flies of Economic Importance, 10–15 September 2006, Salvador, Brazil; 2006. p. 285–293.

[CIT0067] Woods B , Lacey IB, Brockway CA, Johnstone CP. Hosts of Mediterranean fruit fly *Ceratitis capitata* (Wiedemann) (Diptera: Tephritidae) from Broome and the Broome Peninsula, Western Australia. Aust J Entomol. 2005:44(4):437–441. 10.1111/j.1440-6055.2005.00484.x

[CIT0068] Wright C , Wyatt P, Mayer D, Leach P. Improved statistical methods for estimating infestation rates in quarantine research when hosts are naturally infested. J Econ Entomol. 2023:116(6):1990–1997. 10.1093/jee/toad203 37904594 PMC10711534

[CIT0069] Yee WL , Goughnour RB, Hood GR, Forbes AA, Feder JL. Chilling and host plant/site-associated eclosion times of western cherry fruit fly (Diptera: Tephritidae) and a host-specific parasitoid. Environ Entomol. 2015:44(4):1029–1042. 10.1093/ee/nvv097 26314048

